# Development of Na_0.5_CoO_2_ Thick Film Prepared by Screen-Printing Process

**DOI:** 10.3390/ma13122805

**Published:** 2020-06-22

**Authors:** Akihiro Tsuruta, Miki Tanaka, Masashi Mikami, Yoshiaki Kinemuchi, Yoshitake Masuda, Woosuck Shin, Ichiro Terasaki

**Affiliations:** 1 National Institute of Advanced Industrial Science and Technology (AIST), Shimo-Shidami, Moriyama-ku, Nagoya 463-8560, Japan; tanaka.miki@aist.go.jp (M.T.); m-mikami@aist.go.jp (M.M.); y.kinemuchi@aist.go.jp (Y.K.); masuda-y@aist.go.jp (Y.M.); w.shin@aist.go.jp (W.S.); 2 Department of Physics, Nagoya University, Furo-cho, Chikusa-ku, Nagoya 464-8602, Japan; terra@nagoya-u.jp

**Keywords:** screen print, sodium cobaltite, thermoelectric, thick film

## Abstract

The Na_0.5_Co_0.9_Cu_0.1_O_2_ thick film with the same thermoelectric performance as a Na_0.5_CoO_2_ bulk was formed on an alumina substrate by the screen-printing process. The power factor exceeded 0.3 mW/K^2^m, with the resistivity of 3.8 mΩcm and the thermopower of 108 μV/K. The thick film without any cracks strongly adhered to the substrate. The high-quality thick film had been realized through the carefully designed and improved process, mixing NaCl to promote the anisotropic sintering of Na_0.5_Co_0.9_Cu_0.1_O_2_, inserting a CuO interlayer to adhere the film and substrate, and Co–Cu substituting Cu for Co to control the sintering temperature.

## 1. Introduction

Thermoelectric (TE) materials can convert heat energy directly into electrical energy without any moving parts or emitting environmental pollutants. This clean power generation is expected to be a technology that can effectively utilize the waste heat from other power generations as well as various devices, because TE can generate power even from a small amount of thermal energy that cannot turn the turbine. In order to enhance the conversion efficiency, various inorganic materials, such as the Bi-Te system [1,2,3], SnSe [4,5], and Heusler compounds [6,7,8], have been studied steadily for a long time. Recently, flexible and/or wearable devices contributing to IoT have attracted attention, even in the field of thermoelectricity [9], and organic thermoelectric materials such as PEDOT:PSS (Poly(3,4-ethylenedioxythiophene):Poly(styrenesulfonate)) are also being actively studied [10]. 

Harvesting diluted waste heat requires a module design that can concentrate a heat flow into TE materials, and a planar TE device is one of the ways [11,12]. Such a TE device could potentially replace the battery in IoT devices which need a power of several mW, otherwise the battery should be replaced every month. Furthermore, the planar-shaped device realized by the printing process could be freely designed in size and shape, and would be able to propose an appropriate design for various heat sources. A screen-printing process is the most popular and traditional of the various printing processes, because the low process cost and high productivity are suitable for industrial applications. The process has long been used for conductive circuit patterning, and many pastes for metal thick films are commercially available. Even today, the processes and pastes are investigated to realize fine line patterning for the product miniaturization and integration [13,14], and the latest research has achieved printing with a width of 30 μm or less using silver paste [15]. The development of carbon-based conductive circuit printing for flexible devices is also active [16,17], but there are few reports on ceramic material printing, with a serious problem in thick film sintering such as a cracking due to the sintering shrinkage [18,19]. 

We chose oxides as the thermoelectric material, favoring the high stability and low toxicity. The high thermoelectric performance of the metallic layered transition-metal oxide, Na_0.5_CoO_2_, as a p-type TE material had been reported by Terasaki et al. [20]. The low resistivity originates in its high carrier density, whereas the high thermopower is maintained by the orbital and spin entropies derived from the Mott insulator. The thermoelectric power factor S^2^*ρ*^−1^, where *ρ* is resistivity and S is thermopower, of a Na_0.5_CoO_2_ single crystal reaches 5 mW/K^2^m at 300 K in the in-plane direction [20] and exceeds 4 mW/K^2^m of Bi_2_Te_3_ [21]. Since Na_0.5_CoO_2_ shows anisotropic electrical conductivity and the resistivity in the out-of-plane direction is approximately 50 times higher than that in the in-plane direction, the power factor of Na_0.5_CoO_2_ polycrystalline sample is 0.25 mW/K^2^m at 300 K. However, Na_0.5_CoO_2_ demonstrates superior performance compared to the other oxide materials, and is a promising thermoelectric material.

In this paper, we report the thermoelectric properties and structure of developed Na_0.5_CoO_2_ thick film and discuss the significance of our improved process. In particular, we have introduced a sintering additive into the thick film and an interlayer between the thick film and a substrate, and further adjusted the composition of the thick film.

## 2. Experimental 

The Na_0.5_Co_0.9_Cu_0.1_O_2_ powder was prepared by a solid-state reaction. Na_2_CO_3_, Co_3_O_4_, and CuO were mixed and calcined at 860 ℃ for 12 h in air. The calcined products were ground, pressed into a pellet, and sintered at 920 ℃ for 12 h in air. The Na_0.5_Co_0.9_Cu_0.1_O_2_ powder was obtained via a mechanical grinding and ball milling of the pellet. The Na_0.5_CoO_2_ powder was prepared by the same process from Na_2_CO_3_ and Co_3_O_4_.

The paste for the main Na_0.5_Co_0.9_Cu_0.1_O_2_ layer was comprised of a mixture of synthesized Na_0.5_Co_0.9_Cu_0.1_O_2_ powder and ground NaCl powder, a vehicle of Terpineol and Ethyl cellulose. The volume fraction of NaCl in the mixture was optimized to 20 vol%. The paste for the CuO interlayer consisted of CuO powder and the same vehicle as that of the main layer. The pastes of Na_0.5_CoO_2_ without NaCl, and Na_0.5_CoO_2_ with 20 vol% NaCl, were also prepared by mixing the same vehicle.

The CuO paste was printed on an alumina substrate (10 mm × 20 mm × 1 mm) in a rectangular shape (10 mm × 2 mm) using a screen-printing process. The printed film was sintered at 950 ℃ for 12 h in air after drying the vehicle at 200 ℃ for 5 min. Subsequently, the NaCl-mixed Na_0.5_Co_0.9_Cu_0.1_O_2_ paste was printed on the sintered CuO interlayer in the same shape. The vehicle was dried in the same manner as mentioned. The screen printing of both layers was performed at the print speed of 50 mm/s using a stainless steel mesh screen with 250-mesh/inch by a semi-auto screen printing machine (SERIA CORPORATION, SSA-PC150E-IP, Tokyo, Japan). In order to achieve 100 μm of the thickness after drying, the Na_0.5_Co_0.9_Cu_0.1_O_2_ paste was laminated 10 times under the same printing condition. The sintering of the dried film was carried out at 920 ℃ for 1 h in air. 

In this paper, two other kinds of thick films were also prepared for comparison. One is a Na_0.5_CoO_2_ thick film prepared by a simple screen-printing process. Here, only the Na_0.5_CoO_2_ paste was printed and laminated 10 times on an alumina substrate in a rectangular shape (10 mm × 5 mm). Another thick film was prepared by a partially improved process. The NaCl-mixed Na_0.5_CoO_2_ paste was printed and laminated 10 times on a CuO interlayer, which was printed and sintered on an alumina substrate in a rectangular shape (10 mm × 2 mm). The printing, drying, and sintering conditions of both of the main layers (the Na_0.5_CoO_2_ layer and the NaCl-mixed Na_0.5_CoO_2_ layer) and the CuO interlayer were the same with those of the NaCl-mixed Na_0.5_Co_0.9_Cu_0.1_O_2_ layer and the CuO interlayer as mentioned above, respectively.

X-ray diffraction (XRD) patterns of the thick film were taken with CuKα radiation using a standard diffractometer with a monochromator (Rigaku SmartLab, Tokyo, Japan). The XRD measurement was performed in 2*θ*/*θ*-scan mode against the sample surface, i.e., a plane parallel to the substrate surface. The resistivity and thermopower were measured by the four-point probe method and EMFs under temperature gradients using Pt thermocouples, respectively, in air from 50 ℃ to 550 ℃ (Ozawa Science RZ2001S, Nagoya, Japan). The morphology and element mapping of the thick film were observed using a scanning electron microscope (SEM; JEOL, JSM-5600, Tokyo, Japan) and energy-dispersive X-ray spectrometry (EDX; JEOL, EX-54145JMU, Tokyo, Japan). The thermal behaviors of the Na_0.5_CoO_2_ and Na_0.5_Co_0.9_Cu_0.1_O_2_ powders were analyzed by a thermogravimetry and differential thermal analyzer (TG/DTA; BRUKER, MTC1000SA, Billerica, MA, USA) from 15 ℃ to 950 ℃ in air.

## 3. Results and Discussion

### 3.1. Problems with a Thick Film Prepared by a Simple Printing Process

**Figure 1:**
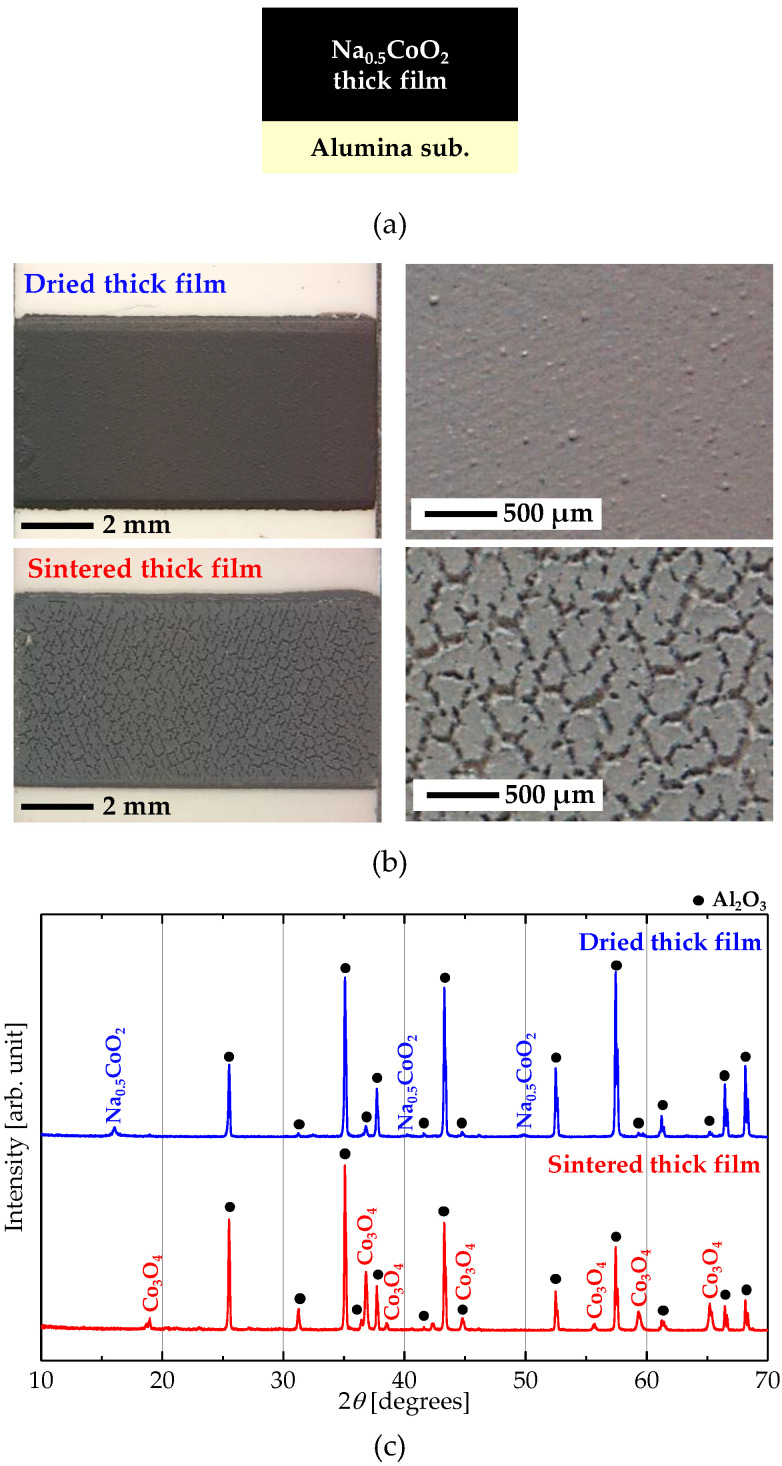
(**a**) Cross-sectional schematic of a printed Na_0.5_CoO_2_ thick film on an alumina substrate as a first approach. (**b**) Photographs and (**c**) XRD (CuKα) patterns of a dried and a sintered Na_0.5_CoO_2_ thick film prepared by the simple screen-printing process.

As a first approach to forming a Na_0.5_CoO_2_ thick film, we simply screen-printed and sintered the Na_0.5_CoO_2_ paste on an alumina substrate. Figure 1a,b show a cross-sectional schematic of a printed thick film, and photographs of a dried and a sintered thick film prepared by the simple screen-printing process, respectively. The thick film was cracked severely after sintering and easily peeled from the substrate. In addition, Na_0.5_CoO_2_ has decomposed to Co_3_O_4_ due to the evaporation of Na during sintering, as shown in Figure 1c. 

**Figure 2:**
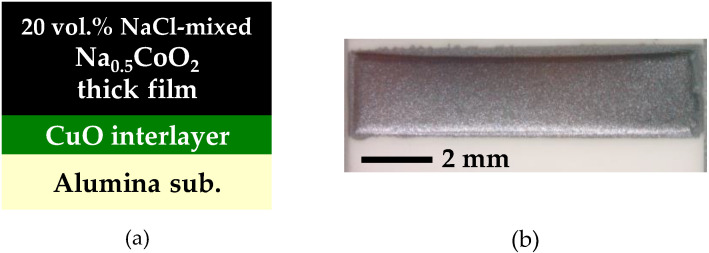
(**a**) Cross-sectional schematic and (**b**) photograph of a partially improved Na_0.5_CoO_2_ thick film with NaCl mixing and a CuO interlayer introduction.

In order to suppress the evaporation of Na, we employed flux sintering using NaCl. Furthermore, the interface between the film and substrate was modified by an insertion of a CuO interlayer in order to improve the adhesion strength. The cross-sectional schematic and the photograph of a partially improved Na_0.5_CoO_2_ thick film with NaCl mixing and a CuO interlayer introduction are shown in Figure 2a,b, respectively. NaCl mixing drastically changes the appearance of the film to be free of cracks, and more importantly, the decomposition was totally prevented. As expected, the CuO interlayer introduction improved the adhesion strength and prevented the peeling of the thick film.

**Figure 3:**
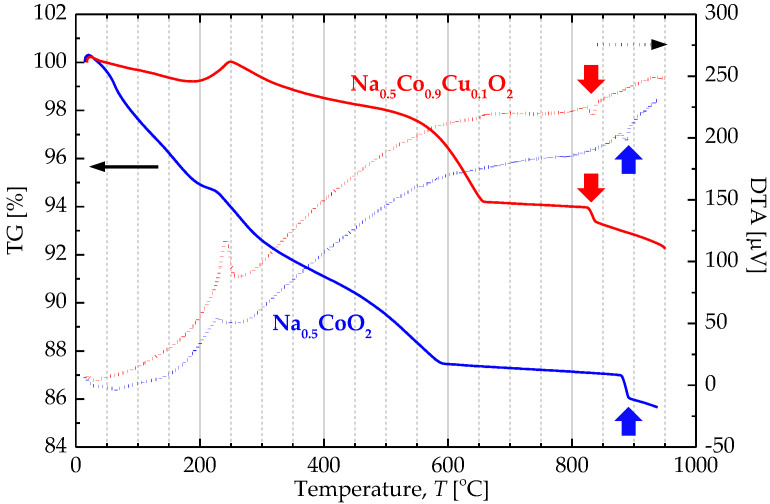
Thermogravimetry/differential thermal analyzer (TG/DTA) curves of Na_0.5_CoO_2_ and Na_0.5_Co_0.9_Cu_0.1_O_2_ powders.

However, bending and arching of the film on the CuO interlayer were found due to the sintering shrinkage. Through several attempts, we found that Cu substitution into Co site prevent the delamination. TG/DTA analysis revealed the difference in the stability between Na_0.5_CoO_2_ and Na_0.5_Co_0.9_Cu_0.1_O_2_ as shown in Figure 3. The weight loss was significantly reduced by the substitution. In addition to that, a faint endothermic peak at 890 ℃ shifted to 830 ℃. K. Park et al. has reported that this Co–Cu substitution reduces the melting temperature of Na_0.5_CoO_2_ [22]. Although the observed peaks are much lower than the melting point, the shift indicates the reduced temperature stability by the substitution as well. In other words, Na_0.5_Co_0.9_Cu_0.1_O_2_ is more reactive than Na_0.5_CoO_2_, which is beneficial to improve sintering. As a result, well-adhered thick film without any cracks was attained as shown later.

### 3.2. Microstructure of Crack-Free Thick Film

**Figure 4:**
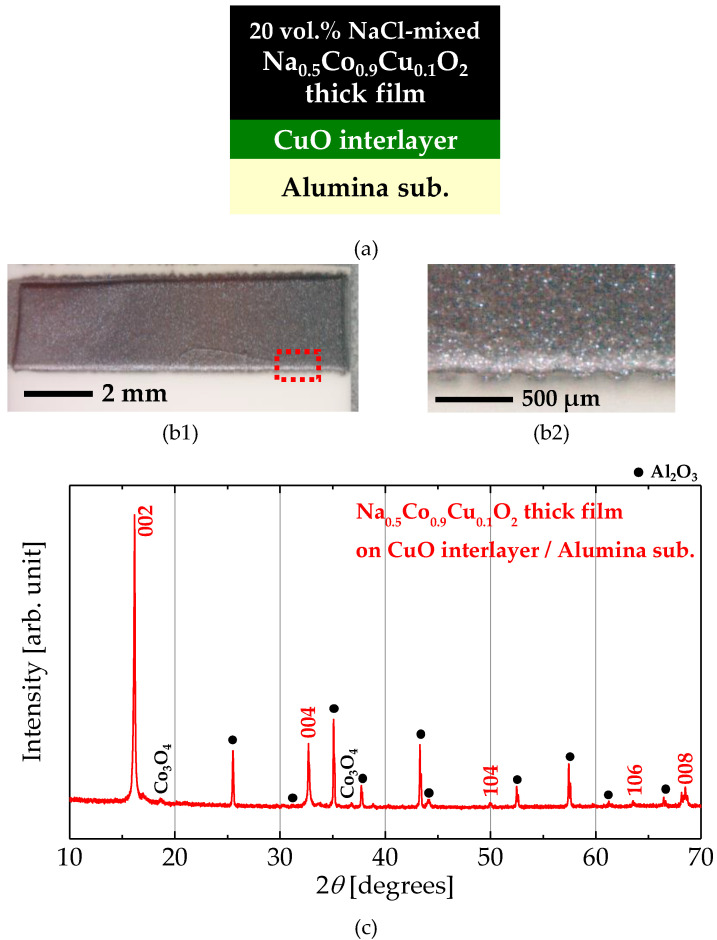
(**a**) Cross-sectional schematic of a Na_0.5_Co_0.9_Cu_0.1_O_2_ thick film prepared by the improved process. (**b1**,**b2**) Photographs of the Na_0.5_Co_0.9_Cu_0.1_O_2_ thick film; (**b1**) shows the entire film and (**b2**) shows an enlarged portion surrounded by a red broken line in (**b1**). (**c**) The XRD (CuKα) pattern of the Na_0.5_Co_0.9_Cu_0.1_O_2_ thick film. The peaks with Miller indices correspond to Na_0.5_CoO_2_ phase.

Figure 4a shows the cross-sectional schematic of the Na_0.5_Co_0.9_Cu_0.1_O_2_ thick film prepared by the improved process. Compared to the partially improved thick film shown in Figure 2a, the composition of the main layer had been changed from Na_0.5_CoO_2_ to Na_0.5_Co_0.9_Cu_0.1_O_2_. Figure 4(b1) shows a photograph of the entire Na_0.5_Co_0.9_Cu_0.1_O_2_ thick film, and Figure 4(b2) shows an enlarged portion of the area surrounded by the red broken line in Figure 4(b1). The thick film has a glossy surface, indicating a finely sintered dense film. No cracks can be seen with an optical microscope. In addition, the thick film is strongly adhered to an alumina substrate and passed the adhesive tape peeling test. This appearance fully satisfies the requirements for a thick film structure and is expected to perform well. 

Figure 4c shows the XRD pattern of the Na_0.5_Co_0.9_Cu_0.1_O_2_ thick film. The indexed peaks correspond to the Na_0.5_CoO_2_ phase. Co_3_O_4_, which is thought to have been formed by the decomposition of a small amount of Na_0.5_Co_0.9_Cu_0.1_O_2_ by the evaporation of Na, is confirmed as an impurity. It should be noted that the Na_0.5_CoO_2_ phase, which was completely degraded in the trial sample, remained mostly in the improved thick film. Since the sintering temperature and atmosphere of the films were exactly the same, the decomposition of the Na_0.5_CoO_2_ phase was suppressed by the Na-rich surroundings with mixed NaCl. The peaks corresponding to the (00*n*) planes of the Na_0.5_CoO_2_ phase are stronger than the other peaks compared to reported powder diffraction patterns. Namely, the *c*-axis of the Na_0.5_CoO_2_ phase is oriented perpendicular to the substrate, at least near the film surface where the X-ray can penetrate. The *c*-axis length, calculated from the 00*n* peaks, are 10.95 Å, and it corresponds to the Na-rich composition of Na_0.68_CoO_2_ in comparison with data reported by Shu et al. [23]. The excess Na, induced from NaCl, has been intercalated into Na_0.5_Co_0.9_Cu_0.1_O_2_. Although the Co–Cu substitution should change the axis length, the effect on the *c*-axis should be slight since the ionic radii of Co and Cu are similar. Certainly, the 00*n* peak shift with the Co–Cu substitution has been invisible in the previous report [22,24].

**Figure 5:**
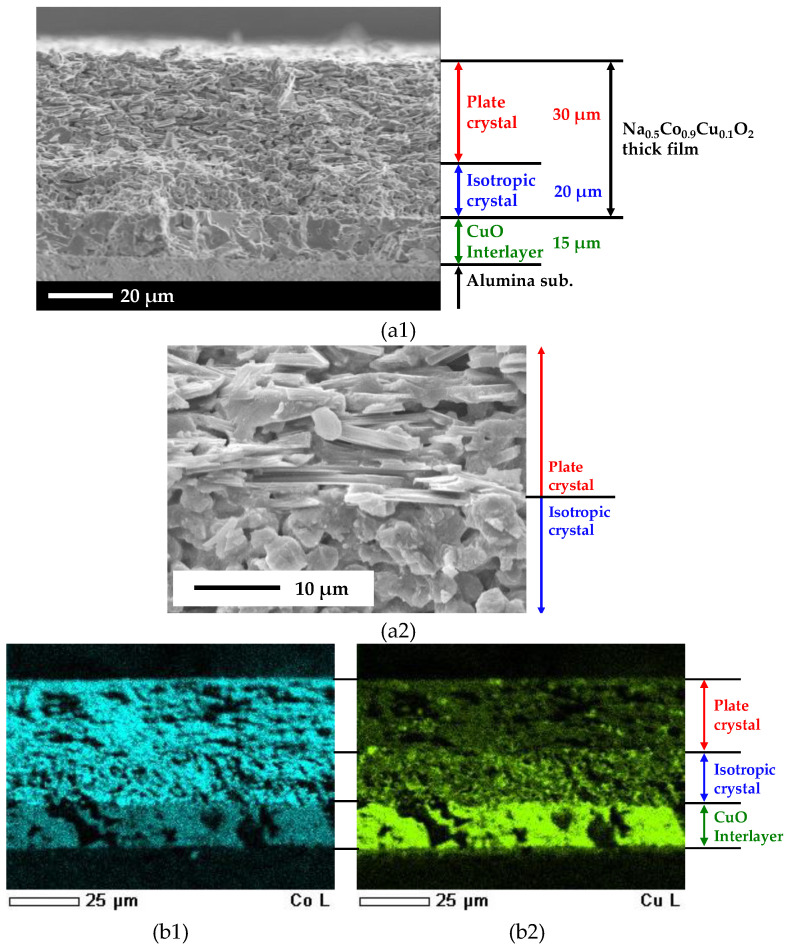
(**a1**) Cross-sectional SEM image of the Na_0.5_Co_0.9_Cu_0.1_O_2_ thick film. (**a2**) An enlarged image of the interface between the plate-crystal layer and the isotropic-crystal layer. (**b1**,**b2**) Element mapping images of the Na_0.5_Co_0.9_Cu_0.1_O_2_ thick film for (**b1**) Co and (**b2**) Cu.

The cross-sectional SEM observation image of the Na_0.5_Co_0.9_Cu_0.1_O_2_ thick film is shown in Figure 5(a1). The well-sintered CuO interlayer with a thickness of approximately 15 μm is clearly observed on the alumina substrate, on which the Na_0.5_Co_0.9_Cu_0.1_O_2_ layer with a thickness of 50 μm is laminated. No cracks can be observed, and all layers are firmly adhered. It is noteworthy that the Na_0.5_Co_0.9_Cu_0.1_O_2_ layer clearly separates a 30 μm-thick plate-crystal layer from the surface and a 20 μm-thick isotropic-crystal layer near the CuO interlayer. Figure 5(a2) shows an enlarged image of the interface between the layers. So far, preferential growth of Na_0.5_CoO_2_ has been reported in the templated grain growth process using NaCl flux [25] as well as hot-forged sintering [26], leading to the well-aligned microstructure along the *ab*-plane. In our Na_0.5_Co_0.9_Cu_0.1_O_2_ thick film, the mixed NaCl would have acted as a flux that promotes the crystal growth of Na_0.5_Co_0.9_Cu_0.1_O_2_. This leads to the growth of plate-crystals, reflecting its layered crystal structure and crystal orientation along with the interface. Typically, cracks in printed thick films are caused by the mismatch between the sintering shrinkage of the film and in-plane constraint from the substrate. Then, brittle ceramic materials are hard to sinter into crack-free thick films (compared to metallic materials that can deform). On the contrary, in our flux sintered thick film, the crystals grew in the in-plane direction of the film, supporting the anisotropic sintering of the film in such a way that effectively suppressed crack formation. Notably, with both the layered crystal structure of Na_0.5_Co_0.9_Cu_0.1_O_2_ and the crystal growth with NaCl flux, the isotropic crystal growth of Na_0.5_Co_0.9_Cu_0.1_O_2_ just above the CuO interlayer is rather unnatural. 

Figure 5(b1,b2) show the cross-sectional element mapping images of the Na_0.5_Co_0.9_Cu_0.1_O_2_ thick film for Co and Cu, respectively. In the plate-crystal layer, Co and Cu coexist, and both are uniformly distributed throughout the layer, indicating that Cu substitutes to Co site in Na_0.5_CoO_2_. On the other hand, in the isotropic-crystal layer, Co is uniformly distributed throughout the layer just as with the plate-crystal layer, but the Cu distribution has a distinct gradation. The high brightness of Cu concentration in the isotropic-crystal layer is approximately the same with the CuO interlayer, suggesting the presence of Cu-rich impurities. Co is also found in the CuO interlayer. Co can substitute Cu site in CuO, thus Co ions would diffuse into the CuO interlayer during the sintering, and NaCl flux would promote the diffusion. 

**Figure 6:**
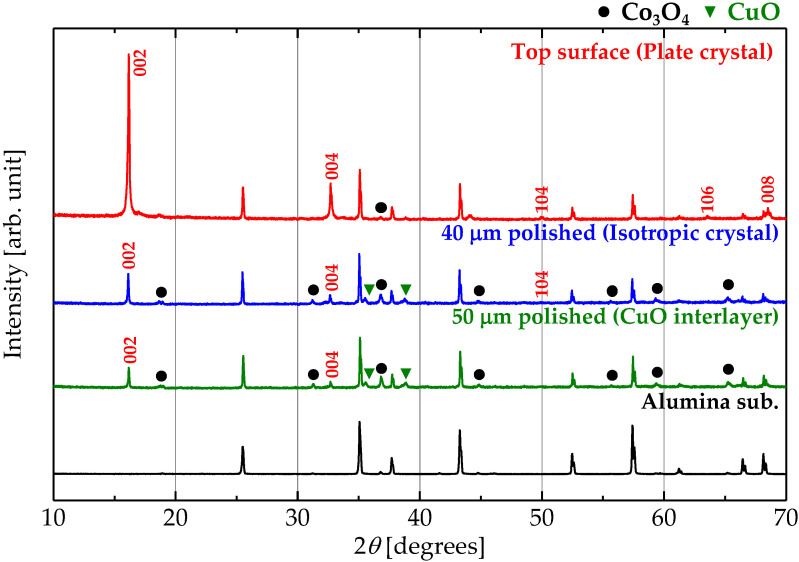
XRD (CuKα) patterns of the plate-crystal layer, the isotropic crystal layer, the CuO interlayer, and the alumina substrate in the Na_0.5_Co_0.9_Cu_0.1_O_2_ thick film. Each layer was exposed by polishing from the surface of the film.

In order to identify the compounds contained in each layer, the thick film was polished from the surface, and each exposed layer was measured by XRD. Figure 6 shows XRD patterns of each layer. The indexed peaks and the unassigned peaks correspond to the Na_0.5_CoO_2_ phase and alumina substrate, respectively. The peaks of the Na_0.5_CoO_2_ phase are located at the same angle in all layers. There are peaks of CuO in addition to the peaks of the Na_0.5_CoO_2_ phase in the isotropic-crystal layer, and Co_3_O_4_ observed in the plate-crystal layer. Hence, the Cu-rich impurity observed in the elemental mapping image is CuO, and the isotropic-crystal layer is a composite of Na_0.5_Co_0.9_Cu_0.1_O_2_, Co_3_O_4_, and CuO. CuO forms large grains because Cu-rich grains are relatively large in the mapping image. Such CuO with a *C12/c1* space group of monoclinic should form isotropic grains and may act as a grain growth inhibitor for Na_0.5_Co_0.9_Cu_0.1_O_2_, resulting in the isotropic Na_0.5_Co_0.9_Cu_0.1_O_2_ crystals. The origin of Cu-source that form CuO grains should be the CuO interlayer exuded as a liquid phase or dissolved in NaCl flux, since the Cu concentration in Na_0.5_Co_0.9_Cu_0.1_O_2_ is much lower than that of CuO. Co in the CuO interlayer formed Co_3_O_4_. 

CuO has been sintered in a liquid phase at an interlayer sintering temperature of 950 ℃, and penetrated into a Na_0.5_Co_0.9_Cu_0.1_O_2_ layer with NaCl flux. Therefore, the CuO interlayer adheres to both of the alumina substrate and the Na_0.5_Co_0.9_Cu_0.1_O_2_ thick film by the anchor effect, and adheres the thick film to the substrate strongly. In addition, CuO is compatible with alumina substrates from the viewpoint of thermal expansion [27,28]. 

### 3.3. Thermoelectric Properties of Na_0.5_Co_0.9_Cu_0.1_O_2_ Thick Film

**Figure 7:**
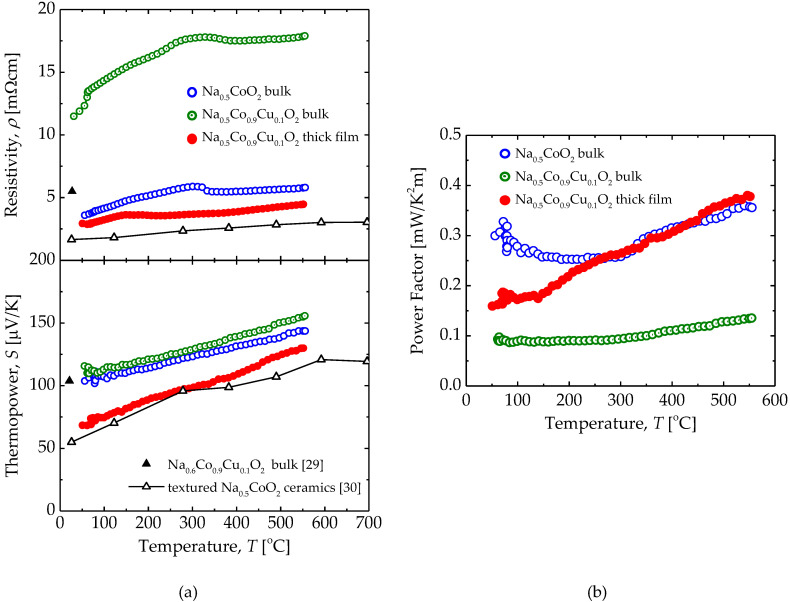
Temperature dependence of (**a**) resistivity, thermopower, and (**b**) power factor for a Na_0.5_CoO_2_ bulk, a Na_0.5_Co_0.9_Cu_0.1_O_2_ bulk, and the Na_0.5_Co_0.9_Cu_0.1_O_2_ thick film measured in air.

Figure 7 shows the temperature (*T*), dependence of resistivity (*ρ*), thermopower (*S*), and power factor (S^2^*ρ*^−1^) of a Na_0.5_CoO_2_ bulk, a Na_0.5_Co_0.9_Cu_0.1_O_2_ bulk, and the Na_0.5_Co_0.9_Cu_0.1_O_2_ thick film. The Na_0.5_CoO_2_ bulk showed similar thermoelectric properties with various reported polycrystalline samples. On the other hand, although the thermopower of our Na_0.5_Co_0.9_Cu_0.1_O_2_ bulk was a similar value to the Na_0.5_Co_0.9_Cu_0.1_O_2_ bulk reported by Terasaki et al. [29], the resistivity was more than twice the reported values [22,29]. The cause of the high resistivity of the bulk should be its low density due to non-optimized process. The Na_0.5_Co_0.9_Cu_0.1_O_2_ thick film shows lower resistivity than our Na_0.5_CoO_2_ bulk and the reported Na_0.5_Co_0.9_Cu_0.1_O_2_ bulk at all temperatures. It has been reported that the resistivity in the in-plane direction is one order of magnitude lower than the resistivity in the out-of-plane direction [20]. Co–Cu substitution and Na content in Na_0.5_CoO_2_ also have an influence on resistivity, because both change the carrier density through reducing the amount, or changing the valence, of Co. However, the change in resistivity caused by the factor related to composition is smaller than that caused by the anisotropy. Hence the preferred orientation of *ab*-plane is responsible for the low resistivity. Certainly, the textured Na_0.5_CoO_2_ ceramics, with the well aligned *ab*-plane prepared by the reactive templated grain growth (RTGG) method [30], show lowest resistivity in Figure 7a. 

The thermopower of the Na_0.5_Co_0.9_Cu_0.1_O_2_ thick film was lower than that of the bulk samples. The thermopower has been reported to be enhanced by a Co–Cu substitution in Na_0.5_CoO_2_, and increased with increasing Na content in a previous study [29,31]. As shown in Figure 7a, Na_0.5_Co_0.9_Cu_0.1_O_2_ bulk shows higher thermopower than the Na_0.5_CoO_2_ bulk. The thermopower of the Na_0.5_Co_0.9_Cu_0.1_O_2_ bulk reported by Park et al. has exceeded 400 µV/K at 600 ℃ [22], and it is much higher than the other reports and the theoretical value calculated from the Heikes formula with the valence ratio of Co^3+^:Co^4+^ = 1:1 [32,33], but the reason for this is not clear. Nevertheless, the Na_0.5_Co_0.9_Cu_0.1_O_2_ thick film, which is expected to be a Na-rich composition with Co–Cu substitution, shows lower thermopower than the Na_0.5_CoO_2_ bulk.

In careful consideration for thermopower reduction, such a low value can only be attributed to the Na-poor composition in the outermost surface of the thick film. Na reduction leads to the increase of the Co^4+^ ratio in Co ions and reduces thermopower, theoretically [34]. A similar unexplained reduction in the thermopower has also been observed in the textured Na_0.5_CoO_2_ ceramics prepared by RTGG method. The thermopower of the Na_0.5_CoO_2_ ceramics and the Na_0.5_Co_0.9_Cu_0.1_O_2_ thick film are in very good agreement. Both have the common features that they are polycrystals with a well aligned *ab*-plane and both have been sintered in a thin form with a large surface area. In both cases, the large surface area might be the cause of the Na-poor composition and thermopower reduction in the outermost surface. 

As a result, the Na_0.5_Co_0.9_Cu_0.1_O_2_ thick film with superior resistivity and inferior thermopower to the Na_0.5_CoO_2_ bulk shows a comparable power factor with Na_0.5_CoO_2_ bulk as shown in Figure 7b.

## 4. Conclusions

We fabricated a Na_0.5_Co_0.9_Cu_0.1_O_2_ thick film with the same thermoelectric performance as a Na_0.5_CoO_2_ bulk on an alumina substrate by screen-printing process. The thick film had no cracks and adhered to the substrate strongly. Mixing NaCl flux into the Na_0.5_Co_0.9_Cu_0.1_O_2_ thick film promoted the sintering of Na_0.5_Co_0.9_Cu_0.1_O_2_ and the formation of plate-crystals according to the anisotropic crystal structure of the Na_0.5_CoO_2_ system. The cracks, which should be formed through the sintering shrinkage, were suppressed via the anisotropic sintering of the thick film caused by the growth and in-plane alignment of the plate Na_0.5_Co_0.9_Cu_0.1_O_2_ crystals. In the Na_0.5_Co_0.9_Cu_0.1_O_2_ thick film, there was an isotropic Na_0.5_Co_0.9_Cu_0.1_O_2_ crystals layer just on the CuO interlayer. CuO inhibited the crystal growth of Na_0.5_Co_0.9_Cu_0.1_O_2_ and made Na_0.5_Co_0.9_Cu_0.1_O_2_ to form isotropic crystals, while providing strong adhesion to the substrate. The *ab*-plane alignment of Na_0.5_Co_0.9_Cu_0.1_O_2_ reduced the resistivity of the thick film compared with a Na_0.5_CoO_2_ bulk. The thick film showed smaller thermopower than a Na_0.5_CoO_2_ bulk. The reason for the thermopower reduction would be Na-poor composition at the outermost surface, and the value and temperature dependence were in very good agreement with the reported Na_0.5_CoO_2_ ceramics with a similar structure. As a result, the Na_0.5_Co_0.9_Cu_0.1_O_2_ thick film with superior resistivity and inferior thermopower to a Na_0.5_CoO_2_ bulk showed a comparable power factor with a Na_0.5_CoO_2_ bulk.
